# MEMORY CARE IN ASSISTED LIVING: REGULATIONS, ACCESS, AND CARE DELIVERY

**DOI:** 10.1093/geroni/igae098.1657

**Published:** 2024-12-31

**Authors:** Paula Carder, Doug Pace

**Affiliations:** Chair; Discussant

## Abstract

Assisted living (AL) is the largest residential care provider of long-term care. Of the nearly one million people who reside in AL, up to 70% have some form of cognitive impairment and 40% have a diagnosis of Alzheimer’s Disease or related dementia (ADRD). AL staff provide services including help with dressing and meals, and nearly 25% of ALs provide specialized care for people living with ADRD (also known as “memory care”). This symposium will include four presentations, each focused on different dimensions of memory care. The first presenter will share findings from a state regulatory review in which the team profiled the presence and content of memory care-related regulations - identifying extreme variability within and across states. The second speaker will present an innovative approach for identifying memory care ALs, and describe their availability across the country. The third presenter will share findings from a recent nationally-representative survey of ALs related to the provision of end of life care in memory care ALs. The fourth speaker will present findings from a national retrospective study in which end of life care outcomes among residents with dementia in memory care will be compared to those in general AL. Finally, our discussant, a national leader in dementia care advocacy and former chair of the Center for Excellence in Assisted Living, will contextualize findings highlighting the perspectives of consumers, AL providers, and policymakers. Assisted Living Interest Group Sponsored Symposium

